# Geographical Residency and Quality of Life in Adults with and without Hearing Loss

**DOI:** 10.1093/geroni/igaa057.3143

**Published:** 2020-12-16

**Authors:** Marcia Hay-McCutcheon, Xin Yang

**Affiliations:** 1 Audiology, Tuscaloosa, Alabama, United States; 2 The University of Alabama, Tuscaloosa, Alabama, United States

## Abstract

There is an increased interest in the impact that hearing loss has on general well-being, including overall quality of life (QOL). The Quality of Life Inventory (QOLI), the Charlson Comorbidity Index and an Accessibility to Health Care questionnaire were administered to 108 participants. For adults with hearing loss who did not have access to hearing health care, lower QOL scores were reported compared to those with access to hearing health care, but this finding was not significant. Effect size calculations indicated that adults with hearing loss who lived in the most rural regions of Alabama, had lower reported QOL scores than their counterparts who had hearing within normal limits. Finally, those with higher incomes, who were older, and who had fewer physical disorders reported higher QOL compared to those with lower incomes, were younger, and who had more physical ailments. Part of a symposium sponsored by the Mental Health Practice and Aging Interest Group.

